# Predictive and Prognostic Effects of Primary Tumor Size on Colorectal Cancer Survival

**DOI:** 10.3389/fonc.2021.728076

**Published:** 2021-12-09

**Authors:** Olatunji B. Alese, Wei Zhou, Renjian Jiang, Katerina Zakka, Zhonglu Huang, Chimuanya Okoli, Walid L. Shaib, Mehmet Akce, Maria Diab, Christina Wu, Bassel F. El-Rayes

**Affiliations:** ^1^ Department of Hematology and Medical Oncology, Winship Cancer Institute, Emory University, Atlanta, GA, United States; ^2^ Winship Data and Technology Applications Shared Resource, Emory University, Atlanta, GA, United States; ^3^ Department of Medicine, Wellstar Atlanta Medical Center, Atlanta, GA, United States; ^4^ Department of Medicine, Advocate Illinois Masonic Medical Center, Chicago, IL, United States

**Keywords:** therapy response determinants, prognostic factors, colorectal cancer, primary tumor size, survival outcomes

## Abstract

**Background:**

Pathologic staging is crucial in colorectal cancer (CRC). Unlike the majority of solid tumors, the current staging model does not use tumor size as a criterion. We evaluated the predictive and prognostic impact of primary tumor size on all stages of CRC.

**Methods:**

Using the National Cancer Database (NCDB), we conducted an analysis of CRC patients diagnosed between 2010 and 2015 who underwent resection of their primary cancer. Univariate and multivariate analyses were used to identify predictive and prognostic factors, Kaplan-Meier analysis and Cox proportional hazards models for association between tumor size and survival.

**Results:**

About 61,000 patients met the inclusion criteria. Median age was 63 years and majority of the tumors were colon primary (82.7%). AJCC stage distribution was: I - 20.1%; II - 32.1%; III - 34.7% and IV - 13.1%. The prognostic impact of tumor size was strongly associated with survival in stage III disease. Compared to patients with tumors <2cm; those with 2-5cm (HR 1.33; 1.19-1.49; p<0.001), 5-10cm (HR 1.51 (1.34-1.70; p<0.001) and >10cm (HR 1.95 (1.65-2.31; p<0.001) had worse survival independent of other variables. Stage II treated without adjuvant chemotherapy had comparable survival outcomes (HR 1.09; 0.97-1.523; p=0.148) with stage III patients who did, while Stage II patients who received adjuvant chemotherapy did much better than both groups (HR 0.76; 0.67-0.86; p<0.001). Stage III patients who did not receive adjuvant chemotherapy had the worst outcomes among the non-metastatic disease subgroups (HR 2.66; 2.48-2.86; p<0.001). Larger tumors were associated with advanced stage, MSI high, non-rectal primary and positive resection margins.

**Conclusions:**

Further studies are needed to clarify the role of tumor size in prognostic staging models, and how to incorporate it into therapy decisions.

## Introduction

Colorectal cancer (CRC) affects about 1.8 million people globally, leading to over 600,000 deaths in 2018 alone and is the second leading cause of cancer-related deaths in the United States (1). Although the median age at diagnosis is 67 years, there is an alarming increase in the number of new cases in young people (2). The 5-year relative survival for patients diagnosed with colorectal cancer is 66% (3). This however varied from 90.6% for localized disease to 14.7% for patients with distant spread.

The American Joint Committee on Cancer (AJCC) tumor (T), nodes (N), and metastases (M) system is a widely recognized cancer risk stratification system used in the management of cancer patients, including colorectal cancer (4, 5). Radiographic and endoscopic assessments are used to assign a clinical stage (cTNM), while resected specimens are evaluated for pathologic stage (pTNM). Patients with rectal cancer, or those with colon primaries treated preoperatively with systemic or radiotherapy are further classified with a post-neoadjuvant pathologic stage (ypTNM). The prognostic significance of the tumor size/horizontal growth of most solid tumors in the TNM staging is reflected by its major role in determining the prognosis, recurrence, survival, and clinical management (6). However, its value in risk stratifying colorectal cancer remains controversial (7, 8). Colorectal cancer spreads horizontally but also invade through the depth of the various layers of the colon wall. The current AJCC colorectal cancer T stage uses depth of tumor invasion rather than tumor size/horizontal spread.

Survival rates unfortunately do not directly correlate with increasing AJCC stage in colorectal cancer. An analysis of the SEER Program public use data file for patients diagnosed in years 1998 to 2000 showed interesting findings. The 2- and 5- year survival rates for patients with AJCC stage I colon cancer were almost identical for those with IIIA. However, stage IIC disease was associated with significantly worse survival rates highlighting the importance of T stage in determining outcome (9). The contribution of tumor size/horizontal growth to the impact of T stage on outcome has not been adequately evaluated. An analysis of 2080 Chinese patients with colorectal carcinoma who underwent surgical resection from 1985 to 2011 compared weighting of the T stage and AJCC for stage homogeneity and discrimination (10). Hazard ratios for both overall and disease specific survival were more accurately predicted by their proposed T-plus staging system which placed extra emphasis on T-stage weighting.

Other retrospective studies have also showed direct correlation between tumor size or maximum horizontal tumor diameter and higher survival in colon cancer patients (11, 12). An analysis of tumor diameter, C-reactive protein concentrations and survival in patients undergoing surgery for colorectal cancer showed no correlation between tumor and increased mortality (13). A more recent study evaluating the association between small node-positive colon cancers with survival in a Military Health System also failed to show differences in overall survival based on tumor size in the study population (14). Although most of these studies were conducted with limited sample sizes (15), the potential for tumor size as a valuable tool in colon cancer survival necessitates additional analyses. This study evaluated the impact of tumor size and horizontal extent on colorectal cancer survival using the largest cohort to date.

## Materials and Methods

The National Cancer Database (NCDB) encompasses about 1500 Commission-on-Cancer-accredited cancer programs and captures more than 70% of all incident cancers in the United States. The aim of this study is to evaluate the impact of tumor size on survival among all CRC patients who underwent surgical resection for their primary tumor between 2010 and 2015. Selection criteria for the study included CRC with pathologic T stage with morphology codes: 8140-47, 8210-11, 8220-21, 8260-63, 8480-81, and 8490. Exclusion criteria were patients with age less than 18 years, missing AJCC pathologic stage, chemotherapy status or preoperative use in non-metastatic disease, tumor size or follow up data, or those treated for additional primary malignancies. The primary outcome was overall survival which was defined as interval between time of diagnosis and death. Patient-specific covariates included age at diagnosis, gender, race, insurance status, year of diagnosis, primary site (colon vs. rectal), AJCC TNM stage, MSI status, treatment received. Ethical approval was based on Emory University IRB waiver policy, since patient information in the database is completely de-identified and the database is legally accessible to the public.

### Statistical Analysis

Tumor size was categorized into three discrete groups: less than 2cm, 2-5 cm, 5-10 cm and greater than 10cm. The demographic, clinical and pathologic characteristics of patients in the study population were summarized using appropriate descriptive statistics for variable type and distribution. Univariate and multivariate analyses were conducted to identify factors associated with patient outcomes. All clinically meaningful variables were included and subsequently eliminated based on the level of significance. To assess the association between patient characteristics and survival, Cox proportional hazards models were fitted with a backward elimination method (removal criteria p=0.05). Likelihood ratio test (LRT) was used to compare the model with the covariate being assessed; both added with the model and with the assessed covariate dropped. An alpha level of 0.05 was used, and any covariate with LRT p-value ˃0.05 was removed from the final multivariate model. Backward elimination was used to automate the LRTs and determine the final model with the covariates presented. Kaplan-Meier curves were generated for overall survival. All statistical analysis was conducted with a significant level of 0.05 using SAS version 9.4 (SAS Institute, Cary NC) and SAS macros developed by the Biostatistics and Bioinformatics Shared Resource at Winship Cancer Institute in Atlanta, Georgia (16).

## Results

### Patient Demographics

A total of 60,999 patients met the eligibility criteria. The mean age at diagnosis was 62.7years (SD+/-14). Gender distribution revealed male were 50.9% (**Table 1**). Majority of the patients were non-Hispanic Whites (82%) and most of the patients had colon primary sites (82.7%). Younger patients (<60 years old) accounted for most of the progressive annual increases in number of CRC cases over the 6 years of study. About 33% of the patients were treated at academic or research cancer centers.

**Table 1:** Demographics of study population.

	AJCC Pathologic Stage Group	Total	I	II	III	IV
Variable	Level	N (%) = 60999	N (%)=12265 (20.1)	N (%)=19565 (32.1)	N (%)=21162 (34.7)	N (%) = 8007 (13.1)
Age	<60	25144 (41.2)	4437 (36.2)	6691 (34.2)	9810 (46.4)	4206 (52.5)
	60-69	15490 (25.4)	3178 (25.9)	4990 (25.5)	5336 (25.2)	1986 (24.8)
	>=70	20365 (33.4)	4650 (37.9)	7884 (40.3)	6016 (28.4)	1815 (22.7)
Sex	Male	31048 (50.9)	6298 (51.3)	9913 (50.7)	10788 (51.0)	4049 (50.5)
	Female	29951 (49.1)	5967 (48.7)	9652 (49.3)	10374 (49.0)	3958 (49.5)
Race	White	50176 (82.3)	10331 (84.2)	16272 (83.2)	17115 (80.9)	6458 (80.6)
	Black	7221 (11.8)	1285 (10.5)	2178 (11.1)	2626 (12.4)	1132 (14.1)
	Others/Unknown	3602 (5.9)	649 (5.3)	1115 (5.7)	1421 (6.7)	417 (5.2)
Primary Payor	Not Insured	2268 (3.7)	261 (2.1)	743 (3.8)	863 (4.1)	401 (5.1)
	Private	27092 (44.4)	5321 (43.4)	7571 (38.7)	10204 (48.2)	3996 (49.9)
	Medicaid/Other Government	4705 (6.7)	582 (4.7)	1200 (6.1)	1526 (7.2)	797 (10.0)
	Medicare	26882 (44.1)	5975 (48.7)	9646 (50.3)	8337 (39.4)	2724 (34.7)
	Unknown	652 (1.1)	126 (1.0)	205 (1.0)	232 (1.1)	89 (1.1)
Median Income Quartiles	<$38,000	9940 (16.3)	1878 (15.3)	3196 (16.3)	3529 (16.7)	1337 (16.7)
	$38,000-$47,999	13381(21.9)	2590 (21.1)	4432 (22.7)	4697 (21.7)	1762 (22.0)
	$48,000-$62,999	16641(27.3)	3408 (27.8)	5177 (26.5)	5843 (27.6)	22213 (27.7)
	$63,000 +	20908 (34.3)	4369 (35.6)	6729 (34.4)	7134 (33.7)	2676 (33.4)
	Not Available	129 (0.2)	20 (0.2)	31 (0.2)	59 (0.3)	19 (0.2)
Year of Diagnosis	2010	6066 (9.9)	1231 (10.0)	1969 (10.1)	2029 (9.6)	837 (10.4)
	2011	8241 (13.5)	1618 (13.2)	2718 (13.9)	2800 (13.2)	1105 (13.8)
	2012	9950 (16.3)	1905 (15.5)	3366 (17.2)	3373 (15.9)	1306 (16.3)
	2013	11244(18.4)	2323 (19.0)	3543 (18.1)	3931 (18.6)	1447 (18.1)
	2014	11985 (19.6)	2411 (19.7)	3766 (19.3)	4243 (20.0)	1538 (19.2)
	2015	13540(22.2)	2777 (22.6)	4203 (21.5)	4786 (22.6)	1774 (22.1)
Primary Site	Colon	50513 (82.7)	9061 (73.8)	17018 (87.0)	173870(82.0)	7064 (88.0)
	Rectum	10486 (17.3)	3204 (26.2)	2547 (13.0)	3792 (18.0)	943 (12.0)
Tumor Size (cm)	0-2cm	8008 (13.1)	4630 (37.7)	1075 (5.5)	1885 (8.9)	418 (5.2)
	2-5cm	31008 (50.8)	6158 (50.2)	9594 (49.0)	11430 (54.0)	35826 (47.8)
	5-10cm	19909 (32.6)	1372 (11.2)	7962 (40.7)	7215(34.1)	3360 (42.0)
	>10cm	2074 (3.4)	105 (0.9)	934 (4.8)	632 (3.0)	403 (5.1)
Surgical Margins Status	Negative	56426 (92.5)	1267 (99.2)	18752 (95.8)	19323 (91.3)	6184 (77.1)
	Positive	4302 (7.1)	70 (0.6)	767 (3.9)	1735 (8.2)	1730 (21.7)
	Unknown	271 (0.4)	28 (0.2)	46 (0.2)	104 (0.5)	93 (1.2)
MSI Status	MSI low	50247 (82.4)	10239 (83.5)	15389 (78.7)	6947 (83.6)	6925 (86.5)
	MSI high	10752 (17.6)	2026 (16.5)	4176 (21.3)	3468 (16.4)	1082 (13.5)
Grade	Well Differentiated	57434 (9.4)	1949 (15.9)	1833 (9.4)	1482 (7.0)	470 (5.9)
	Moderately Differentiated	42924 (68.7)	8840 (72.1)	14209 (72.6)	13992 (66.1)	4883 (61.0)
	Poorly Differentiated / Undifferentiated	11367 (18.6)	998 (8.1)	2975 (15.2)	5063 (23.9)	2331 (29.1)
	Cell Type Not Determined	1974 (3.2)	478 (3.9)	548 (2.8)	625 (3.0)	323 (4.1)
Charlson-Deyo Score	0	43736 (71.7)	8429 (68.7)	13528 (69.1)	15607 (73.7)	6272 (77.1)
	1	12421 (20.4)	2689 (21.9)	4233 (21.6)	4196 (19.4)	1403 (17.5)
	2	3327 (5.4)	766 (6.2)	1226 (6.3)	1014 (4.8)	321 (4.0)
	>=3	1515 (2.5)	381 (3.1)	578 (3.0)	445 (2.1)	111 (1.4)

### Impact of Tumor Size on Clinical Presentation and Tumor Characteristics

The distribution across stages I-III and resected primary for stage IV was 20.1%, 32.1%, 34.7% and 13.2%; respectively. Most of the primary tumors were less than 2-5cm in size (50.8%), followed by 5-10cm (32.6%), <2cm (13.1%) and >10cm (3.4%). Most of the tumors were moderately differentiated (68.7%) and 82% had microsatellite stable (MSS) disease. When compared to smaller primary tumors (less than 2cm in size), those larger than 10cm were more likely to present with advanced disease (stage IV: 19% vs. 5%; p<0.001), have high level microsatellite instability (MSI-H: 32.9% vs. 12.7%; p<0.001), and were less likely to be of rectal origin (9.6% vs. 25.7%; p<0.001) (**Table 2**). They were also more likely to result in positive margins at resection (14% vs. 2.7%; p<0.001) and higher frequency of poorly differentiated histology (33.1% vs. 10.7% p<0.001). There were no obvious disparities in the co-morbidity index (Charlson-Deyo Score) based on tumor size.

**Table 2:** Univariate correlation with tumor size.

			Tumor Size (cm)								p-value	
			0-2cm N=8008		2-5cm N=31008		5-10cm N=19909		>10cm N=2074			
Age N (Col %)		<60	3249 (40.57)		12529 (40.41)		8462 (42.5)		904 (43.59)		**<.001**	
		60-69	2229 (27.83)		7869 (25.38)		4898 (24.6)		494 (23.82)			
		>=70	2530 (31.59)		10610 (34.22)		6549 (32.89)		676 (32.59)			
Sex N (Col %)		Male	4142 (51.72)		15444 (49.81)		10376 (52.12)		1086 (52.36)		**<.001**	
		Female	3866 (48.28)		15564 (50.19)		9533 (47.88)		988 (47.64)	
Race N (Col %)		White	6681 (83.43)		25605 (82.58)		16177 (81.25)		1713 (82.59)		**<.001**	
		Black	842 (10.51)		3505 (11.3)		2612 (13.12)		262 (12.63)	
		Others/Unknown	485 (6.06)		1898 (6.12)		1120 (5.63)		99 (4.77)	
Primary Payor N (Col %)		Not Insured	958 (2.73)		1169 (4.87)		151 (7.26)		151 (7.28)		**<.001**	
		Private	15931 (45.42)		10423 (43.45)		817 (39.26)		814 (39.25)	
		Medicaid/Other Government	2386 (6.8)		2093 (8.73)		227 (10.91)		203 (9.79)	
		Medicare	15449 (44.04)		10026 (41.8)		862 (41.42)		882 (42.53)	
		Unknown	353 (1.01)		276 (1.15)		24 (1.15)		24 (1.16)	
Median Income Quartiles N (Col %)		$38,000	5509 (15.71)		4061 (16.93)		405 (19.46)		402 (19.38)		**<.001**	
		$38,000-$47,999	7565 (21.57)		5360 (22.35)		487 (23.4)		485 (23.38)	
		$48,000-$62,999	9614 (27.41)		6555 (27.33)		511 (24.56)		509 (24.54)	
		$63,000 +	12324 (35.13)		7953 (33.16)		671 (32.24)		671 (32.35)	
		Not Available	65 (0.19)		58 (0.24)		7 (0.34)		7 (0.34)	
Year of Diagnosis N (Col %)		2010	3519 (10.03)		2363 (9.85)		203 (9.75)		202 (9.74)		0.157	
		2011	4790 (13.66)		3195 (13.32)		278 (13.36)		277 (13.36)	
		2012	5703 (16.26)		3905 (16.28)		368 (17.68)		366 (17.65)	
		2013	6526 (18.6)		4356 (18.16)		394 (18.93)		393 (18.95)	
		2014	6845 (19.51)		4776 (19.91)		364 (17.49)		362 (17.45)	
		2015	7694 (21.93)		5392 (22.48)		474 (22.78)		474 (22.85)	
AJCC Pathologic Stage Group N (Col %)		I	4630 (57.82)		6158 (19.86)		1372 (6.89)		105 (5.06)		**<.001**	
		II	1075 (13.42)		9594 (30.94)		7962 (39.99)		934 (45.03)			
		III	1885 (23.54)		11430 (36.86)		7215 (36.24)		632 (30.47)			
		IV	418 (5.22)		3826 (12.34)		3360 (16.88)		403 (19.43)			
Primary Site N (Col %)		Colon	5951 (74.31)		25372 (81.82)		17314 (86.97)		1876 (90.45)		**<.001**	
		Rectum	2057 (25.69)		5636 (18.18)		2595 (13.03)		198 (9.55)	
Surgical Margins Status N (Col %)		Negative	7764 (96.95)		29030 (93.62)		17862 (89.72)		1770 (85.34)		**<.001**	
		Positive	219 (2.73)		1855 (5.98)		1937 (9.73)		291 (14.03)	
		Unknown	25 (0.31)		123 (0.4)		110 (0.55)		13 (0.63)	
MSI Status N (Col %)		MSI low	6991 (87.3)		26506 (85.48)		15359 (77.15)		1391 (67.07)		**<.001**	
		MSI high	1017 (12.7)		4502 (14.52)		4550 (22.85)		683 (32.93)	
Regional Lymph Nodes Positive N (Col %)		Negative	5824 (72.73)		17015 (54.87)		10357 (52.02)		1185 (57.14)		**<.001**	
		Positive	2055 (25.66)		13796 (44.49)		9465 (47.54)		881 (42.48)	
		Not examined	117 (1.46)		171 (0.55)		78 (0.39)		8 (0.39)	
		Unknown	12 (0.15)		26 (0.08)		9 (0.05)		0 (0)	
Grade N (Col %)		Well Differentiated	1326 (16.56)		2781 (8.97)		1486 (7.46)		141 (6.8)		**<.001**	
	Moderately Differentiated		5511 (68.82)		22286 (71.87)		12963 (65.11)		1164 (56.12)	
		Poorly Differentiated/U ndifferentiated	861 (10.75)		5028 (16.22)		4790 (24.06)		688 (33.17)	
		Cell Type NotDetermined	310 (3.87)		913 (2.94)		670 (3.37)		81 (3.91)	
Charlson-Deyo Score	0		5722 (71.45)		22114 (71.32)		14413 (72.39)		1487 (71.7)		**0.003**	
	1		1654 (20.65)		6363 (20.52)		3962 (19.9)		442 (21.31)	
N (Col %)	2		426 (5.32)		1724 (5.56)		1073 (5.39)		104 (5.01)	
	>=3		206 (2.57)		807 (2.6)		461 (2.32)		41 (1.98)	
Chemotherapy	Yes		3080 (38.46)		17882 (57.67)		12433 (62.45)		1307 (63.02)		**<.001**	
N (Col %)	No		4928 (61.54)		13126 (42.33)		7476 (37.55)		767 (36.98)	

Values in bold were statistically significant.

Stratification by AJCC stage showed the majority tumor size with stage I disease were 2-5cm (50.2%) followed by <2cm (37.8%), and less than 1% larger than 10cm (p<0.001). In contrast, only 5.2% of stage IV cancers presented with tumor size <2cm, with 47% larger than 5cm (compared to about 13% in stage I; p<0.001). More than half of stage IV disease presentation (55%) were in patients younger than 60 years of age, compared with 22.6% in patients who were 70 years or older (p<0.001). However, almost equal number of patients <60 years (36.2%) and >70 years (37.9%) presented with stage I disease (p<0.001). African Americans presented with more stage IV disease compared to non-Hispanic whites (16% vs. 12.9%; p<0.001). Rectal primaries were also more likely to present with early disease stage; 26.2% at stage I compared to 11.8% at stage IV (p<0.001). Most patients who underwent surgical removal of stage I disease had negative resection margins (99.2%) compared to stage IV disease (77%; p<0.001). Stage I disease was also less likely to have poorly differentiated histology (8.1%) compared to stages II, II or IV; 15.1%, 23.9 and 29% respectively (p<0.001). Although limited in number, the proportion of rectal cancer patients with positive surgical margins was double those of colon cancer for stage I disease (0.5% vs. 1%; p<0.001).

There were 19,565 patients with stage II disease (pT3 or 4, N0). Tumor size was similarly distributed between 2-5cm (49%) and 5-10cm (40.7%). Fewer patients had tumors <2cm in size (5.5%) or >10cm (4.8%). The patients also had majority primary colon location (86.9%) and about 33.6% received chemotherapy. Stage III disease (Any T, N+) was diagnosed in 21,162 patients. Tumor size distribution was <2cm (8.9%), 2-5cm (54%), 5-10cm (34.1%) and >10cm (3%). Compared to stage II patients, there was a lower frequency of MSI-H disease (16.4% vs. 21.3%). There was also a higher incidence of poorly differentiated histology (23.9% vs. 15.2%).

We identified 8,007 patients with stage IV CRC who underwent surgical resection and pathologic AJCC T-staging of their primary tumor. About 34% were treated at Academic or Research Programs. Most patients were younger than 70 years of age (77.4%). Primary tumor size distribution was <2cm (5.2%), 2-5cm (47.8%), 5-10cm (42%) and >10cm (5.1%). Majority of the patients had Charlson-Deyo Score of 0 or 1 (94.6%) and there were negative regional LNs (Any T, N0, M1) in 20.1% of the patients. Among patients with tumors >10cm, 7.6% had rectal primary location compared to 13.7% for those with tumors <5cm (p<0.001). Patients with tumors >10cm were also more likely to have positive resection margins (28.7%) compared to those with tumors <5cm (18.6%; p<0.001).

### Impact of Tumor Size on Survival

**Figure 1:**
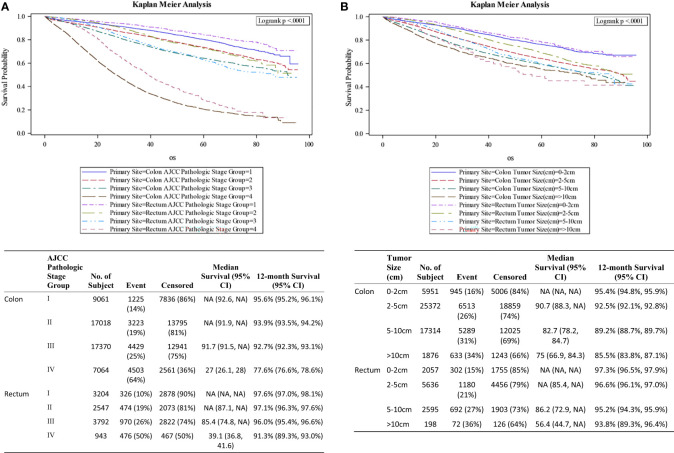
**(A)** Survival curves by stage. **(B)** Survival curves by tumor size.

**Figure 1A** shows the survival curves for both colon and rectal cancer patients across all stages while differential survival based on tumor size alone is displayed in **Figure 1B**. Univariate analysis showed statistically significant correlation between survival and smaller tumor size, age younger than 60 years, male gender, private insurance, higher socioeconomic status, lower AJCC pathologic stage, rectal primary location, negative surgical margin, well differentiated histology, lower Charlson-Deyo score and lack of chemotherapy (**Table 3**). On multivariate analysis, AJCC stage correlated closely with OS (HR 1.44, 2.21, 8.10 for stages II to IV compared to stage I) (**Table 4**). When compared to patients with tumor size <2cm, the prognostic impact of tumor size alone for the whole study population was significant for 2-5cm (HR 1.20; 1.13-1.28; p<0.001), 5-10 cm (HR 1.38; 1.30-1.48; p<0.001) and >10cm (HR 1.55; 1.41-1.71; p<0.001).

**Table 3:** Univariate association with overall survival. REF- Reference.

Covariate	Level	N	Last Contact or Death, Months from Dx		
			----------------------------------------		
			Hazard Ratio (95% CI)	HR P-value	Log-rank P-value
Tumor Size (cm)	2-5cm	31008	1.67 (1.57-1.77)	**<.001**	**<.001**
	5-10cm	19909	2.15 (2.02-2.29)	**<.001**	
	>10cm	2074	2.60 (2.37-2.85)	**<.001**	
	0-2cm	8008	REF	**-**	
Age	60-69	15490	1.21 (1.16-1.26)	**<.001**	**<.001**
	>=70	20365	2.01 (1.94-2.08)	**<.001**	
	<60	25144	REF	**-**	
Sex	Female	29951	0.94 (0.91-0.97)	**<.001**	**<.001**
	Male	31048	REF	**-**	
Race	Black	7221	1.05 (1.00-1.10)	0.062	**<.001**
	Others/Unknown	3602	0.78 (0.73-0.84)	**<.001**	
	White	50176	REF	**-**	
Primary Payor	Not Insured	2268	1.43 (1.32-1.56)	**<.001**	**<.001**
	Medicaid/Other Government	4105	1.59 (1.49-1.70)	**<.001**	
	Medicare	26882	1.86 (1.80-1.93)	**<.001**	
	Unknown	652	1.52 (1.30-1.77)	**<.001**	
	Private	27092	REF	**-**	
Median Income Quartiles	<$38,000	9940	1.32 (1.26-1.38)	**<.001**	**<.001**
	$38,000-$47,999	13381	1.24 (1.19-1.30)	**<.001**	
	$48,000-$62,999	16641	1.13 (1.08-1.17)	**<.001**	
	Not Available	129	1.25 (0.88-1.78)	0.212	
	$63,000 +	20908	REF	**-**	
AJCC Pathologic Stage Group	II	19565	1.52 (1.43-1.61)	**<.001**	**<.001**
	III	21162	2.15 (2.04-2.28)	**<.001**	
	IV	8007	7.34 (6.93-7.77)	**<.001**	
	I	12265	REF	**-**	
Primary Site	Rectum	10486	0.76 (0.72-0.79)	**<.001**	**<.001**
	Colon	50513	REF	**-**	
Surgical Margins Status	Positive	4302	3.23 (3.09-3.37)	**<.001**	**<.001**
	Unknown	271	2.17 (1.81-2.60)	**<.001**	
	Negative	56426	REF	**-**	
MSI Status	MSI high	10752	1.01 (0.97-1.05)	0.768	0.769
	MSI low	50247	REF	**-**	
Regional Lymph Nodes Positive	Positive	26197	2.18 (2.11-2.25)	**<.001**	**<.001**
	Not examined	374	1.72 (1.41-2.08)	**<.001**	
	Unknown	47	1.46 (0.85-2.51)	0.176	
	Negative	34381	REF	**-**	
Grade	Moderately Differentiated	41924	1.09 (1.03-1.16)	**0.004**	**<.001**
	Poorly Differentiated/Undifferentiated	11367	2.03 (1.91-2.17)	**<.001**	
	Cell Type Not Determined	1974	1.17 (1.05-1.29)	**0.005**	
	Well Differentiated	5734	REF	**-**	
Charlson-Deyo Score	1	12421	1.29 (1.24-1.34)	**<.001**	**<.001**
	2	3327	1.76 (1.65-1.86)	**<.001**	
	>=3	1515	2.30 (2.13-2.49)	**<.001**	
	0	43736	REF	**-**	
Chemotherapy	Yes	34702	1.22 (1.18-1.26)	**<.001**	**<.001**
	No	26297	REF	**-**	

Values in bold were statistically significant.

REF, References.

**Table 4:** Multivariable survival analysis stratified by tumor size.

Covariate	Level	N	Last Contact or Death, Months from Dx		
			----------------------------------------		
			Hazard Ratio (95% CI)	HR P-value	Type3 P-value
Tumor Size (cm)	2-5cm	31008	1.20 (1.13-1.28)	**<.001**	**<.001**
	5-10cm	19909	1.38 (1.30-1.48)	**<.001**	
	>10cm	2074	1.55 (1.41-1.71)	**<.001**	
	0-2cm	8008	REF	-	
Age	60-69	15490	1.20 (1.14-1.26)	**<.001**	**<.001**
	>=70	20365	1.96 (1.85-2.07)	**<.001**	
	<60	25144	REF	-	
Sex	Female	29951	0.87 (0.84-0.90)	**<.001**	**<.001**
	Male	31048	REF	-	
Race	Black	7221	0.98 (0.93-1.04)	0.553	**0.029**
	Others/Unknown	3602	0.90 (0.83-0.97)	**0.008**	
	White	50176	REF	-	
Primary Payor	Not Insured	2268	1.34 (1.23-1.46)	**<.001**	**<.001**
	Medicaid/Other Government	4105	1.36 (1.27-1.45)	**<.001**	
	Medicare	26882	1.28 (1.22-1.34)	**<.001**	
	Unknown	652	1.23 (1.05-1.44)	**0.009**	
	Private	27092	REF	-	
Median Income Quartiles	<$38,000	9940	1.26 (1.20-1.33)	**<.001**	**<.001**
	$38,000-$47,999	13381	1.17 (1.12-1.22)	**<.001**	
	$48,000-$62,999	16641	1.08 (1.03-1.12)	**<.001**	
	Not Available	129	0.93 (0.65-1.33)	0.688	
	$63,000 +	20908	REF	-	
AJCC Pathologic Stage Group	II	19565	1.44 (1.35-1.53)	**<.001**	**<.001**
	III	21162	2.21 (2.03-2.42)	**<.001**	
	IV	8007	8.10 (7.44-8.82)	**<.001**	
	I	12265	REF	-	
Surgical Margins Status	Positive	4302	1.98 (1.89-2.07)	**<.001**	**<.001**
	Unknown	271	1.76 (1.47-2.11)	**<.001**	
	Negative	56426	REF	-	
MSI Status	MSI high	10752	0.91 (0.88-0.95)	**<.001**	**<.001**
	MSI low	50247	REF	-	
Regional Lymph Nodes Positive	Positive	26197	1.59 (1.49-1.69)	**<.001**	**<.001**
	Not examined	374	1.65 (1.35-2.01)	**<.001**	
	Unknown	47	1.48 (0.86-2.56)	0.157	
	Negative	34381	REF	-	
Grade	Moderately Differentiated	41924	1.01 (0.95-1.07)	0.705	**<.001**
	Poorly Differentiated / Undifferentiated	11367	1.47 (1.37-1.57)	**<.001**	
	Cell Type Not Determined	1974	1.02 (0.92-1.14)	0.677	
	Well Differentiated	5734	REF	-	
Charlson-Deyo Score	1	12421	1.19 (1.14-1.24)	**<.001**	**<.001**
	2	3327	1.55 (1.46-1.65)	**<.001**	
	>=3	1515	2.13 (1.97-2.31)	**<.001**	
	0	43736	REF	-	
Chemotherapy	Yes	34702	0.50 (0.47-0.52)	**<.001**	**<.001**
	No	26297	REF	-	

*Number of observations in the original data set = 60999. Number of observations used = 60999.

**Backward selection with an alpha level of removal of .20 was used. The following variables were removed from the model: Primary Site.

Values in bold were statistically significant.

REF, References.

Age younger than 60 years was associated with improved survival compared to those 70 years or older (HR 1.91; 1.85-2.07; p<0.001). And patients in the lowest median income quartiles (<$38,000) also had inferior survival outcomes compared to those with $63,000 or more (HR 1.26; 1.20-1.33; p<0.001). Other co-variates associated with improved outcomes included chemotherapy received (HR 0.50; 0.47-0.52; p<0.001), female gender (HR 0.87; 0.84-0.90; p<0.001) and MSI-H status (HR 0.92; 0.88-0.95; p<0.001). Worse outcomes were associated with positive resection margins (HR 1.98; 1.89-2.07; p<0.001), regional lymph node involvement (HR 1.59; 1.49-1.69; p<0.001), poorly differentiated histology (HR 1.47; 1.37-1.57; p<0.001) and Charlson-Deyo Score of 3 or greater (HR 2.13; 1.97-2.31; p<0.001).

Among patients with stage II disease, there were no statistically significant differences between survival outcomes for tumor size <2cm compared to those 2-5cm (HR 0.99; 0.86-1.15; p=0.942), 5-10cm (HR 1.04; 0.90-1.21; p=0.583) or >10cm (HR 1.17; 0.96-1.44; p=0.123). Co-variates associated with improved outcomes in these patients included chemotherapy received (HR 0.73; 0.66-0.80; p<0.001), female gender (HR 0.83; 0.78-0.89; p<0.001) and MSI-H status (HR 0.80; 0.73-0.87; p<0.001). The following were associated with worse outcomes - positive resection margins (HR 2.54; 2.23-2.89; p<0.001), age 70 years or older (HR 2.73; 2.42-3.08; p<0.001), poorly differentiated histology (HR 1.27; 1.11-1.45; p<0.001) and Charlson-Deyo Score of 3 or greater (HR 2.36; 2.06-2.71; p<0.001).

Stage III patients had differential survival rates. Compared to those with tumor size less than 2cm, those with tumors 2-5cm (HR 1.33; 1.19-1.49; p<0.001), 5-10cm (HR 1.51; 1.34-1.70; p<0.001) and >10cm (HR 1.95; 1.65-2.31; p<0.001) had worse survival. Co-variates associated with improved outcomes included chemotherapy received (HR 0.33; 0.31-0.36; p<0.001) and female gender (HR 0.83;0.78-0.87; p<0.001). Co-variates associated with worse outcomes were positive resection margins (HR 2.39; 2.21-2.57; p<0.001), age 70 years or older (HR 1.67;1.52-1.83; p<0.001), poorly differentiated histology (HR 1.61;1.43-1.81; p<0.001) and Charlson-Deyo Score of 3 or greater (HR 1.97; 1.70-2.27; p<0.001). MSI-H status was not statistically associated with survival (HR 0.95; 0.88-1.02; p=0.148).

**Figure 2:**
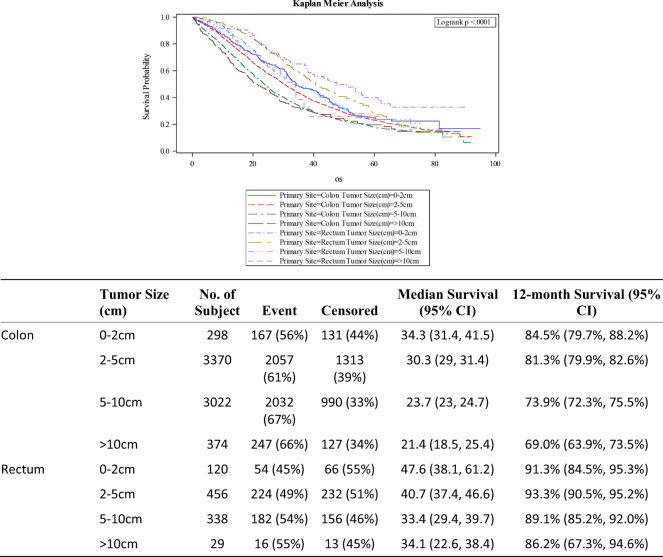
Overall survival for Stage IV CRC stratified by tumor size.

The median overall survival among Stage IV colon cancer patients was stratified by tumor size: 34.3 months for tumor size <2cm; 30.3 months for 2-5cm; 23.7 months for 5-10cm and 21.4 months for tumors larger than 10cm (**Figure 2**). Corresponding mOS values for rectal cancer were higher at 47.6, 40.7, 33.4 and 34.1 months respectively. The survival advantage for rectal primary tumors was statistically significant (HR 0.82; 0.74-0.90; p<0.001). Other co-variates associated with improved outcomes on multivariate analysis included treatment at Academic or Research Programs (HR.85; 0.78-0.92; p<0.001) and use of chemotherapy (HR 0.43; 0.40-0.47; p<0.001). Co-variates associated with worse outcomes were positive resection margins (HR 1.59; 1.50-1.70; p<0.001), age 70 years or older (HR 1.54;1.40-1.70; p<0.001), poorly differentiated histology (HR 1.60;1.41-1.82; p<0.001), lower median income (<38,000 – HR 1.19; 1.10-1.30; p<0.001), and Charlson-Deyo Score of 3 or greater (HR 1.46; 1.17-1.83; p<0.001).

### Correlation Between Tumor Size and Use of Chemotherapy

**Figure 3:**
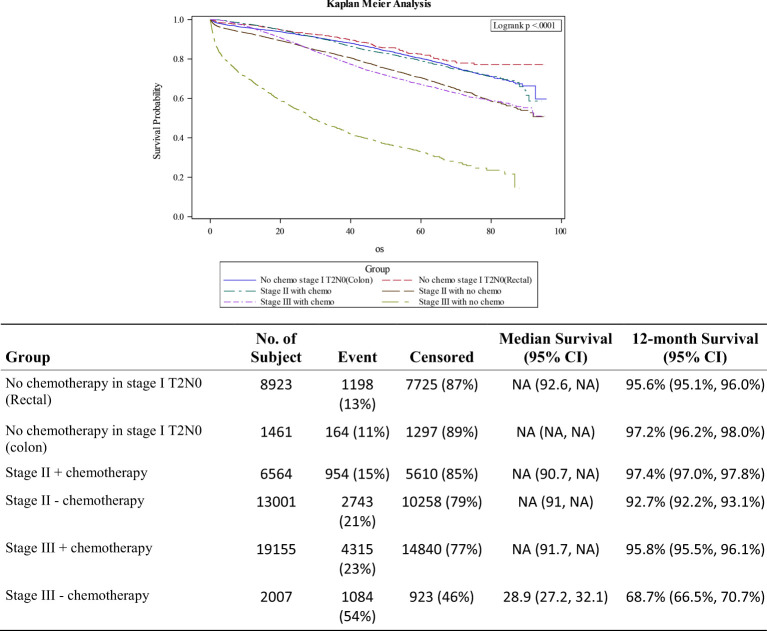
Survival curves for stage I colon/rectal cancers, stage II CRC +/- adjuvant therapy and stage III after adjuvant chemotherapy.

Stage II was stratified into patients who did (n=6564) or did not (n=13,001) receive adjuvant chemotherapy and compared them with patients with stage III CRC who did (n=19,155) or did not receive adjuvant chemotherapy (n=2.007). Tumor size was not taken into account for determination of adjuvant therapy, and use was equally distributed among the 4 categories; <2cm, 2-5cm, 5-10cm and >10cm. Patients 70 years and older with stage II disease were less likely to receive adjuvant chemotherapy (18.3% vs. 51.4%; p<0.001). Patients with Charlson-Deyo Score of 2 or greater were also less likely to receive adjuvant therapy (5% vs. 11.3%; p<0.001). Stage II treated without adjuvant chemotherapy had comparable survival outcomes (HR 1.09; 0.97-1.523; p=0.148) with stage III patients who did, while Stage II patients who received adjuvant chemotherapy did much better than both groups (HR 0.76; 0.67-0.86; p<0.001). Stage III patients who did not receive adjuvant chemotherapy had the worst outcomes among the non-metastatic disease subgroups (HR 2.66; 2.48-2.86; p<0.001). **Figure 3** shows the various survival curves for stage I colon and rectal cancer patients, and stages II and III CRC treated with or without adjuvant chemotherapy. Patients with stage III disease who did not receive chemotherapy had the worst overall survival outcomes, a 12-month overall survival rate of 68.7%, compared to 95.8% for those who did.

## Discussion

CRC remains a significant cause of cancer morbidity and mortality in the US. The current study is the largest to evaluate the impact of tumor size on survival in CRC. Our study population had comparable demographics to previous studies (10, 13). African Americans and patients younger than 60 years of age presented with more advanced disease, reinforcing racial/age disparities that have been previously well documented (2, 17–19). A relatively high proportion (33%) of the patients were treated at academic or research cancer centers. Overall, most primary tumors were less than 5cm in size, and only 3.4% were larger than 10cm. They were however associated with traditionally poor prognostic factors such as advanced or stage IV disease, positive margins at resection and poorly differentiated histology. With the increasing adoption of screening, fewer patients are presenting with metastatic disease (20). The more frequent diagnosis at earlier stages and lower occurrence at rectal location could be due to earlier development of symptoms (21, 22). This analysis confirmed the favorable impact of earlier stage at diagnosis, higher socioeconomic index and microsatellite instability (MSI-H). The availability of remarkably effective immunotherapy for MSI-H tumor subtypes has revolutionized the landscape of cancer therapy, including in patients with metastatic colorectal cancer (23–25).

Different studies have evaluated the pattern of colorectal cancer recurrence based on tumor size (26, 27). Using 1538 patients with stage I CRC to risk stratify metastatic potential and recurrence, maximal tumor size > 5 cm was associated with increased incidence of recurrence among other variables (28). Although the prognostic impact of tumor size was relatively mild in our overall study population, significant findings were observed for different AJCC stages. Compared to smaller tumors, size >10cm in patients with stages I-IV was associated with worst survival rates independent of other variables. Stage IV patients in our study population who underwent resection of their primary tumors were more likely to have less morbidity index (Charlson-Deyo Score of 0 or 1: 94.6%). Those with tumors >10cm were more likely to have colon primary location and positive resection margins. Their inferior median overall survival (mOS) at 22.6 months is remarkable, compared to 32.9 months for tumors <5cm which is the benchmark in most recent literature (29, 30). Further studies exploring measures to reduce survival differences based on tumor sizes are urgently needed.

This study confirmed the use of adjuvant or perioperative chemotherapy as a good prognostic factor. Although tumor size is generally not taken into account for determination of adjuvant therapy, there was equal distribution of its use among the 4 categories of tumor sizes: <2cm, 2-5cm, 5-10cm and >10cm. Remarkably, correcting for tumor size showed no survival difference between stages II and III patients who received adjuvant chemotherapy. Stage II CRC patients without traditional high-risk features (pT4, bowel obstruction or perforation, positive resection margins/lymphovascular/perineural invasion, poorly differentiated histology or less than 12 lymph nodes resected) are generally observed on surveillance, although there is evidence that adjuvant chemotherapy may be beneficial (31, 32). This study showed that tumors greater than 10cm are associated with worse outcomes.

The use of adjuvant chemotherapy in patients with tumors greater than 10cm may explain comparable survival rates irrespective of lymph node status. We found that correcting for tumor sizes showed no statistically significant survival differences between stage I colon and rectal cancer patients (monitored post resection with observation only) and stage III patients who received adjuvant chemotherapy. Findings from a similar study of the SEER database were validated using the databases of the Fudan University Shanghai Cancer Center (6). The authors demonstrated a significant predictive ability of tumor size in T1 colon cancer, outperforming all other factors used in clinical practice. It would be interesting to evaluate the SEER cohort of colon cancer patients with incongruent survival outcomes per AJCC stages, for the impact of tumor sizes on survival after adjuvant chemotherapy.

The retrospective design of this study is an important limitation due to inherent biases. Despite careful curation of the NCDB of most cancer patients in the US, various variables such as specific chemotherapy or systemic agent utilized, disease free survival, recurrence rates, objective assessment of response to treatment and prior history of malignancies (33). Further, missing molecular characteristics such as BRAF mutation have been established as major determinants of therapy response and survival in colorectal cancer (34). Neoadjuvant or preoperative therapy is typically used in rectal cancers with clinical stage T3N0 and greater. The consequent downstaging can be difficult to account for and may explain the smaller rectal primary tumor size. Finally, there is always a possibility that selection bias and tumor sidedness (left vs. right) could have contributed to the survival disparities observed in our study. Right sided are generally larger and associated with delayed presentation of symptoms. They are also more likely to harbor BRAF mutations. Notwithstanding these limitations, our findings have important implications. A prospective interventional study with tumor size as a determinant of neoadjuvant or adjuvant therapy is crucial to verify the findings of our study and improve survival in CRC patients. This may allow patients with Stage II CRC without traditional high-risk features to receive systemic therapy in a controlled manner with a view to improving the currently suboptimal intermediate and long-term survival outcomes.

In conclusion, tumors larger than 10cm have inferior outcomes among patients in the same AJCC stages. We reported that stage II patients who did not receive adjuvant chemotherapy did worse than stage III who did. Therefore, it is imperative to determine the role of adjuvant chemotherapy based on tumor size in patients with node negative disease, who may be at substantial risk for recurrence or metastatic spread despite the absence of traditional high-risk features. Finally, prospective studies are urgently needed to clarify the role of tumor size in staging models for choice of optimal management.

## Data Availability Statement

Publicly available datasets were analyzed in this study. This data can be found here: https://www.facs.org/quality-programs/cancer/ncdb/publicaccess.

## Ethics Statement

Ethical review and approval were not required for the study on human participants in accordance with the local legislation and institutional requirements. The ethics committee waived the requirement of written informed consent for participation.

## Author Contributions

Conceptualization: OA, RJ, and BE-R. Formal analysis: OA, WZ, KZ, RJ, ZH, and BE-R. Investigation: OA, WZ, KZ, RJ, ZH, CO, WS, MA, MD, CW, and BE-R. Methodology: OA, WZ, KZ, RJ, ZH, and BE-R. Writing - original draft, review and editing: OA, WZ, KZ, RJ, ZH, CO, WS, MA, MD, CW, and BE-R. All authors contributed to the article and approved the submitted version.

## Funding

Research reported in this publication was supported in part by the Winship Research Informatics Shared Resource of Winship Cancer Institute of Emory University and NIH/NCI under award number P30CA138292. The data used in the study are derived from a de-identified NCDB file. The NCDB is a joint project of the Commission on Cancer of the American College of Surgeons and the American Cancer Society. The American College of Surgeons and the Commission on Cancer have not verified and are not responsible for the analytic or statistical methodology employed, or the conclusions drawn from these data by the investigators.

## Conflict of Interest

The authors declare that the research was conducted in the absence of any commercial or financial relationships that could be construed as a potential conflict of interest.

## Publisher’s Note

All claims expressed in this article are solely those of the authors and do not necessarily represent those of their affiliated organizations, or those of the publisher, the editors and the reviewers. Any product that may be evaluated in this article, or claim that may be made by its manufacturer, is not guaranteed or endorsed by the publisher.
